# Association between synovial fluid levels of aggrecan ARGS fragments and radiographic progression in knee osteoarthritis

**DOI:** 10.1186/ar3217

**Published:** 2010-12-31

**Authors:** Staffan Larsson, Martin Englund, André Struglics, L Stefan Lohmander

**Affiliations:** 1 Department of Orthopedics, Clinical Sciences Lund, Lund University, BMC C12, Klinikgatan 28, SE-221 84 Lund, Sweden; 2 Clinical Epidemiology Research & Training Unit, Boston University School of Medicine, 650 Albany Street, Suite X200, Boston, MA 02118, USA

## Abstract

**Introduction:**

Aggrecanase cleavage at the ^392^Glu-^393^Ala bond in the interglobular domain (IGD) of aggrecan, releasing N-terminal ^393^ARGS fragments, is an early key event in arthritis and joint injuries. We determined whether synovial fluid (SF) levels of ARGS-aggrecan distinguish subjects with progressive radiographic knee osteoarthritis (ROA) from those with stable or no ROA.

**Methods:**

We studied 141 subjects who, at examination A, had been given meniscectomies an average of 18 years earlier (range, 15 to 22 years). Seventeen individuals without surgery, and without known injury to the menisci or cruciate ligaments, were used as references. At examinations A and B, with a mean follow-up time of 7.5 years, we obtained SF and standing tibiofemoral and skyline patellofemoral radiographs. SF ARGS-aggrecan was measured with an electrochemiluminescence immunoassay, and we graded radiographs according to the OARSI atlas. The association between SF ARGS levels at examination A and progression of radiographic features of knee OA between examinations A and B was assessed by using logistic regression adjusted for age, gender, body mass index, and time between examinations, and stratified by ROA status at examination A.

**Results:**

We found a weak negative association between SF ARGS concentrations and loss of joint space: the likelihood of progression of radiographic joint space narrowing decreased 0.9 times per picomole per milliliter increase in ARGS (odds ratio (OR) 0.89; 95% confidence interval (CI), 0.79 to 0.996). In subjects with and without preexisting ROA at examination A, the association was OR, 0.96; 0.81 to 1.13; and 0.77; 0.62 to 0.95, respectively. Average levels of SF ARGS 18 years after meniscectomy were no different from those of reference subjects and were not correlated to radiographic status at examination A.

**Conclusions:**

In subjects with previous knee meniscectomy but without ROA, levels of SF ARGS-aggrecan were weakly and inversely associated with increased loss of joint space over a period of 7.5 years.

## Introduction

In osteoarthritis (OA), the balance between cartilage-matrix synthesis and degradation is disturbed, resulting in a gradual destruction of the articular cartilage [1]. Collagen type II and aggrecan are the two major constituents of the matrix, and their proteolysis is regarded as a critical event in joint disease [2-12]. In mouse models of OA, collagenolysis by matrix metalloprotease-13 [11], and aggrecanolysis by aggrecanase-2 [5,6], were proven crucial for development of disease. Aggrecanolysis may be a prerequisite for collagenolysis [13]. Molecular fragments resulting from these degradative processes appear in synovial fluid (SF), blood, and urine, and have been investigated as biomarkers for diagnosis, disease severity, onset, or progression [14-16]. The clinical diagnosis of OA relies on symptoms in combination with radiographic changes, both of which appear late in the disease process; molecular biomarkers are being tested for an earlier detection of the disease. Urinary levels of C-telopeptides of type II collagen (CTX-II) have, for example, been shown to be associated with both the presence and the progression of radiographic hip and knee OA [17]. Proteolytic aggrecan fragments are early markers of joint-matrix damage [13], and increased levels of proteoglycan in SF were reported in acute injury and acute inflammatory arthritis [18-21]. We showed with an assay specific for the aggrecanase-generated ARGS neoepitope that SF levels of ARGS-aggrecan are increased in human knee disease, and that measurements of this neoepitope better discriminate between health and disease than do aggrecan levels determined by methods not specific for proteolytic cleavage [10,12]. The hypothesis of the present study was that an association exists between SF ARGS and the development of radiographic knee OA in a cohort of individuals after meniscectomy [22-24].

## Materials and methods

### Subjects

**Figure 1:**
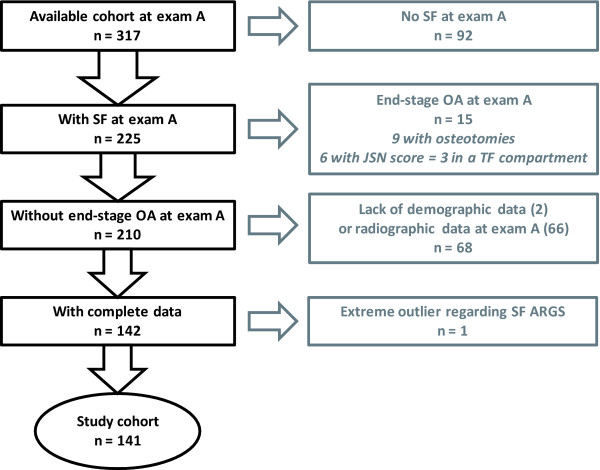
**Flowchart of inclusion and exclusion of subjects in the study**.

The study was approved by the ethics committee of the Faculty of Medicine at Lund University; informed consent was obtained from all participants. Subjects were from a cohort of 317 patients, retrospectively identified to have undergone isolated meniscectomy at Lund University Hospital in 1973, 1978, or between 1983 and 1985 [23]. The first examinations (A) were performed in 1994, 1995, and 2000, respectively, and the second examination (B), in 2004. The mean time from meniscectomy to examination A was 18 years, and the mean time between examinations A and B was 7.5 years (Table 1). As described [23], reasons for exclusion were previous knee surgery, meniscectomy in both knee compartments, osteochondritis dissecans, fracture in or adjacent to the knee, septic arthritis, osteonecrosis, any ligament injury, or radiographic signs of knee OA at the time of surgery. Of 859 identified subjects, 456 fulfilled criteria and were invited to participate at examination A; 329 responded, and 317 had radiographs taken. Here we further excluded subjects with end-stage OA (defined subsequently) of the index knee at examination A. Lack of SF or radiographic or demographic data at examination A also were reasons for exclusion. In all, 141 of the available 317 subjects were included in this study (Figure 1, Table 1). These 141 subjects were studied as one group, or stratified for absence or presence of radiographic OA (ROA) at examination A to address possible floor or ceiling effects on the SF ARGS levels created by the quantity or quality (or both) of the cartilage in the joint.

**Table 1:** Characteristics of the study subjects

	No stratification	Stratified ± ROA at examination A		References
			
	*n *= 141 (100%)	-ROA, *n *= 63 (45%)	+OA, *n *= 78 (55%)	*n *= 17 (100%)
Men	116 (82%)	49 (78%)	67 (86%)	15 (88%)
Age at examination A, years	51 (31-73)	51 (32-73)	52 (31-73)	54 (37-70)
BMI at examination A, kg/m^2^	26 (18-41)	26 (18-35)	27 (21-41)	26 (20-31)
Years between index surgery and examination A	18 (15-22)	18 (15-22)	18 (15-22)	Na
Years between examinations A and B	7.5 (4.0-10.4)	7.1 (4.0-10.1)	7.8 (4.0-10.4)	8.6 (8.6-8.8)
ROA at examination A	78 (55%)	0 (0%)	78 (100%)	1 (6%)
ROA at examination B	106 (75%)	28 (44%)	78 (100%)	1 (6%)
End-stage OA at examination B	31 (22%)	2 (3.2%)	29 (37%)	0 (0)
Arthroplasty or osteotomy at examination B	2 (1.4%)^a^	0 (0)	2 (2.6%)^a^	0 (0)
Loss of joint space/Progression of the JSN score	76 (54%)	22 (35%)	54 (69%)	2 (12%)
Progression of osteophytes	66 (47%)	21 (33%)	45 (58%)	2 (12%)
Progression of ROA	98 (70%)	35 (56%)	63 (81%)	4 (24%)
SF ARGS at examination A, pmol ARGS/ml	6.95 (0.15-15.07)	6.96 (0.15-14.58)	6.94 (0.31-15.07)	Nd
SF ARGS at examination B, pmol ARGS/ml	Nd	Nd	Nd	7.19 (3.63-12.72)

^a^One arthroplasty and one osteotomy. Na, not applicable; Nd, not determined. Values are expressed as numbers (%) or mean (range). BMI, body mass index; JSN, joint space narrowing; OA, osteoarthritis; ROA, radiographic OA.

### Reference group

Seventeen individuals from a previously described reference group with no known knee injury had SF at examination B and complete radiographic data, and were included as references (Table 1) [23].

### Radiographic examination

At examination A, we obtained standing anteroposterior radiographs of the tibiofemoral (TF) joint in about 15 degrees of flexion and a skyline view of the patellofemoral (PF) joint with the knee in about 50 degrees of flexion by using a fluoroscopically positioned x-ray beam, by using film [24]. At examination B, a digital x-ray sensor was used, and posteroanterior and lateral views of the TF joint obtained by using the fixed flexion (SynaFlexer) protocol [25,26].

Joint space narrowing (JSN) and osteophytes in the TF and PF joints were graded on a 4-point scale (0 to 3, where 0 = no evidence of JSN or osteophytes) according to, and in comparison with, images provided in the 1995 atlas of Osteoarthritis Research Society International (OARSI) [27]. Two investigators blinded to clinical data each graded all paired radiographs with knowledge of the time sequence. Images were reread with adjudication of discrepancies between the investigators.

#### Sum scores of JSN, osteophytes and radiographic osteoarthritis

The sum of all JSN or osteophyte grades of an index knee were termed the JSN score or osteophyte score; the sum of all JSN and osteophyte grades in combination was termed the radiographic osteoarthritis score (ROA).

#### Radiographic osteoarthritis score

A knee was defined as having ROA with any of the following scores, according to the 1995 atlas of OARSI [27]:

1. JSN in any TF compartment or the PF compartment of grade 2 or higher.

2. Osteophyte score in the medial or lateral TF compartment or the PF compartment of 2 or more.

3. JSN grade 1 and osteophyte grade 1 in *the same *TF compartment or JSN grade 1 and osteophyte grade 1 in the PF compartment.

This cut-off approximates grade 2 TF OA on the Kellgren and Lawrence (K/L) scale [24].

#### End-stage osteoarthritis

A knee was considered to have end-stage ROA either (a) with JSN grade 3 in any of the TF compartments or in the PF compartment, or (b) when a subject had undergone subsequent tibial osteotomy or arthroplasty for OA.

#### Progression of radiographic features of osteoarthritis

We considered progression of the radiographic features of OA--loss of joint space, progression of osteophytes, or progression in either or both of those features (termed progression of ROA)--to have occurred with an increase from examination A to examination B of the JSN score, the osteophyte score, or their sum, respectively, by 1 or more in any of the TF compartments or the PF compartment. This includes both incident JSN or osteophytes at examination B and worsening of already existing changes.

### Materials

Chemicals were as described [28]. Human recombinant ADAMTS-4 (a disintegrin and metalloproteinase with thrombospondin motifs; aggrecanase-1) [29], and monoclonal antibody (MAb) OA-1, specific for the N-terminal ARGS neoepitope generated by aggrecanase cleavage at the Glu-Ala bond within the aggrecan interglobular domain [30], were from GlaxoSmithKline (Collegeville, PA, USA). MAb AHP0022 against human aggrecan, described as specific for the hyaluronic acid-binding region (HABR) by the manufacturer, and as binding to both G1 and G2 of human aggrecan according to others [31], was from Invitrogen (Carlsbad, CA, USA). Chondroitinase ABC (EC 4.2.2.4), keratanase (EC 3.2.1.103), and keratanase II (from *Bacillus *sp. Ks36) were from Seikagaku (Tokyo, Japan). High-bind MA600 96-well microtiter plates (no. L11XB-1), streptavidin with Sulfo-Tag (streptavidin tagged with the reporter molecule ruthenium(II) *tris*-bipyridyl, no. R32AD), 4× Read Buffer T with surfactant (no. R92TC), and the Sector Imager 6000 with software Discovery Workbench 2006 MSD_3_0_18 were from Meso Scale Discovery (MSD, Gaithersburg, MD, USA).

### Treatment of SF samples and standard

Knee SF was centrifuged at 3,000 *g *for 10 minutes at room temperature, and the supernatant was stored at -80°C. Twenty-five microliter aliquots of SF samples were deglycosylated for 3 hours at 37°C in a final volume of 32.5 μl by using 0.4 mU chondroitinase ABC and keratanase, and 0.02 mU keratanase II per microliter SF in 50 m*M *Tris, 50 m*M *sodium acetate, 10 m*M *EDTA, 1 m*M *AEBSF, and 10 m*M *NEM, at pH 7.3. ARGS standard was made by complete ADAMTS-4 digestion of human aggrecan, which was extracted from knee cartilage by 4 *M *guanidinium hydrochloride and purified by cesium chloride density-gradient centrifugation by using the A1D1 fraction, with a subsequent deglycosylation with chondroitinase ABC (3 mU/μg), keratanase (1 mU/μg), and keratanase II (0.1 mU/μg), as described [28].

### Measurement of ARGS-aggrecan by aggrecan capture OA-1 ARGS electrochemiluminescence (ELCL) assay

SF levels of aggrecan fragments containing the ARGS neoepitope were analyzed by using electrochemiluminescence (ELCL) technology on the Meso Scale Discovery (MSD) platform [32-34].

High-bind 96-well microtiter plates were coated overnight at 4°C with 25 μl/well of anti-human aggrecan (AHP0022) diluted to 60 μg/ml in PBST (0.01 *M *sodium phosphate, 0.138 *M *sodium chloride, 0.0027 *M *potassium chloride, 0.05% Tween 20; pH 7.4). After a wash (all washes 3 × 400 μl PBST), plates were blocked for 1 hour at 22°C with 150 μl/well of PBST containing 1% wt/vol BSA and 1% wt/vol nonfat dry milk. Plates were washed and incubated (2 hours, 22°C, plate shaker) with 25 μl/well of duplicates of standards (2.5 to 0.0073 pmol ARGS/ml) or SF (final dilutions, 1:4 to 1:26) diluted in PBST containing 1% wt/vol BSA. After a wash, plates were incubated, as described earlier, with 25 μl/well of 1 μg/ml biotinylated anti-ARGS (OA-1) and 1 μg/ml Sulfo-Tagged streptavidin in PBST containing 1% wt/vol BSA. After a final wash, 150 μl/well of 4× Read Buffer diluted 1:2 in Millipore water was added, and plates were read in a Sector Imager 6000. Sample concentrations of ARGS-aggrecan were calculated from the standard curve (four-parameter logistic) by using the MSD software. A control SF was deglycosylated and plated in duplicates on each plate and used for inter- and intraassay precision. Seven SFs were spiked after deglycosylation with equimolar concentrations of standard and analyzed in the ARGS ELCL assay. To assess agreement between the ARGS ELCL assay and a published ARGS ELISA by using the same capture antibody (AHP0022) but a different detection antibody (BC-3; Abcam, Cambridge, UK) [35], 43 SF samples from a cross-sectional cohort, spanning a wide range of ARGS concentrations and diagnoses (eight with acute inflammatory arthritis, 35 with acute or chronic knee injuries) previously analyzed in the ARGS ELISA were analyzed in the ARGS ELCL assay.

### Western blot

To test the specificity of the AHP0022 anti-human aggrecan antibody, 2.4 μg of human aggrecan standard was deglycosylated, reduced, and separated on a 3% to 8% Tris-acetate gel, transferred, and probed with AHP0022 (1:500) or MAb OA-1 (1:2,000) by using peroxidase-conjugated horse anti-mouse IgG (CST, Danvers, MA; 1:25,000) as secondary antibody, or with anti-G1 (PA1-1747; Affinity BioReagents, Golden, CO; 1:400) with peroxidise-conjugated goat anti-rabbit IgG (KPL, Gaithersburg, MD; 1:75,000), as described [28].

### Statistical analysis

We used Pearson's correlation (*r*) for continuous variables and Spearman's rank order correlation (*r*_S_) when categoric variables were included. We found SF ARGS to be normally distributed, as tested with Shapiro-Wilks (*P *= 0.093). For group comparisons, we used analysis of covariance (ANCOVA); comparison of SF ARGS in male and female subjects were calculated with and without adjustments for age, BMI, and time between meniscectomy and examination A. Longitudinal associations between SF ARGS at examination A and progression of radiographic features of knee OA were assessed by using univariate and multivariate logistic regression. Odds ratios (ORs) with adjustments for age, gender, BMI, and time between examinations A and B, and respective crude ORs were calculated to estimate the likelihood for progression of ROA. Progression was defined as both incident ROA and worsening of already existing ROA at examination A. We also performed analyses of radiographic progression in knees with or without ROA at examination A separately. We considered a value of *P *< 0.05 to be significant. All tests were two-tailed and performed by using PASW Statistics (SPSS, Chicago, IL, USA) for Windows, version 17.0.3.

## Results

### Technical performance of the aggrecan capture OA-1 ARGS ELCL assay

**Figure 2:**
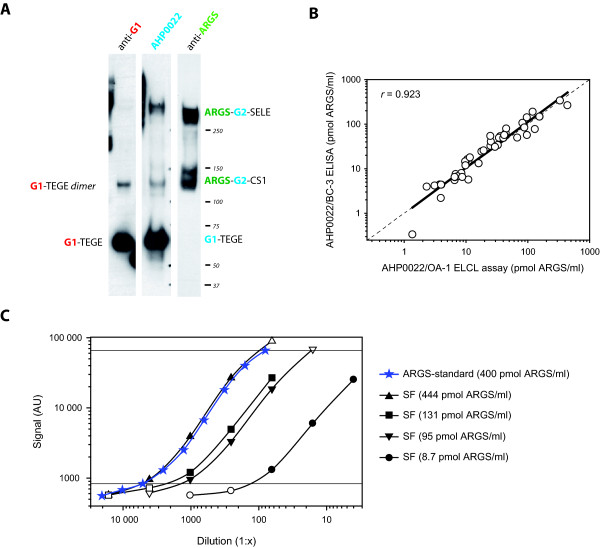
**Specificity and technical performance of the aggrecan capture OA-1 ARGS ELCL assay**. **(a) **2.4 μg of ADAMTS-4-digested and deglycosylated human aggrecan was separated on a 3 to 8% Tris-acetate gel, transferred and probed with anti-G1, anti-aggrecan (AHP0022), or anti-ARGS (OA-1). Protein standard molecular weights and aggrecan fragments detected are indicated. Epitopes recognized by the antibodies are written in color: red, anti-G1; blue, anti-aggrecan (AHP0022); green, anti-ARGS. Aggrecan domains and amino acid sequences: G1 and G2, globular domains 1 and 2; CS1 and CS2, chondroitin sulfate-rich domains 1 and 2; TEGE and ARGS: C- and N-terminal amino acid sequences at the aggrecanase cleavage site within the interglobular domain; SELE, C-terminal amino acid sequence of aggrecanase cleavage within the CS2 [1]. **(b) **ARGS concentration measured with the OA-1 ARGS ELCL assay and the BC-3 ARGS ELISA [35] in 43 individual SFs with a regression line, dashed line of equality, and Pearson's correlation coefficient (*r*). **(c) **Dilution curves of ARGS-aggrecan standard and four synovial fluids (SFs) analyzed in the OA-1 ARGS ELCL assay. Sample concentrations of ARGS-aggrecan at different dilutions were calculated from the standard curve (four-parameter logistic). Dilutions falling within the range of detection of the standard curve (solid symbols within horizontal lines) were used for linearity of dilution calculations (Table 2), with mean values presented in the legend.

In Western blot analysis of the ADAMTS-4 digested human aggrecan used as ARGS-standard, the aggrecan antibody AHP0022 reacts with both G1-containing fragments corresponding to G1-TEGE fragments, and with the two major ARGS fragments, ARGS-SELE and ARGS-chondroitin sulfate-rich domain 1 (CS1), which both contain the G2 domain but not the G1 domain (Figure 2a). As noted by others [31], this indicates that the AHP0022, described by the manufacturer as specific for the hyaluronic acid-binding region (HABR) of aggrecan, recognizes an epitope present in both the G1 and G2 domains, which share homology [36].

Chondroitinase ABC, keratanase, and keratanase II digestion was necessary at 0.4, 0.4, and 0.02 mU/μl SF, respectively. Lower concentrations or exclusion of any of the three enzymes resulted in decreased signal, and the addition of a hyaluronidase digestion before the described deglycosylation had no effect on the signal (not shown). With SFs diluted 1:4 or more, dilution curves of four SFs were parallel to the standard curve within the same range of ARGS concentration per well as the standard curve (Figure 2c). Table 2 details the technical performance of the ARGS ELCL assay. The mean difference between results obtained with ELISA and ELCL was 0.71 pmol/ml (Figure 2b).

**Table 2:** Technical performance of the aggrecan capture OA-1 ARGS ELCL assay

Range of detection of standards	0.075 to 5 pmol ARGS/ml
Minimum required dilution of SF	1:4
Lower limit of detection in SF	0.3 pmol ARGS/ml SF
Parallelism (obtained/expected) of four SF samples diluted 1:4 to 1:4,000^a^	100% (78% to 120%)^b^
Spiking recovery of equimolar spiking of seven SF samples	116% (92% to 133%)^b^
Intra-assay CV, *n *= 14	3.5%
Inter-assay CV, *n *= 9	16.6%

CV, coefficient of variation; ELCL, electrochemiluminescence; SF, synovial fluid. ^a^SFs with 9, 95, 131, and 444 pmol ARGS/ml diluted and read within the range of detection of the standard curve. ^b^Mean (range).

### Patient characteristics, radiographic status, and SF levels of ARGS-aggrecan

At a mean age of 51 years at examination A, 78 (55%) of 141 subjects had ROA 18 years after meniscectomy. At examination B 7.5 years later, 28 additional subjects had ROA, or 106 (75%) of 141 (Table 1). This was a considerably higher proportion than that in the reference subjects, of whom one (6%) of 17 had ROA at both examination A and at examination B 8.6 years later.

The SF ARGS levels at examination A in the 141 subjects after meniscectomy were normally distributed and ranged from 0.15 to 15.07 pmol/ml, with a mean of 6.95 pmol/ml. This includes one sample of a subject who had a concentration below the level of detection that was assigned a value of 0.15 pmol ARGS/ml (that is, half the lower limit of detection). One sample of a subject had an extreme level of ARGS (31 pmol/ml), which was more than 7 times the interquartile range and was excluded.

The average level and range of SF ARGS in the 68 individuals excluded from the study because of lack of demographic or radiographic data (Figure 1) did not differ from those observed in the included subjects (not shown).

No SF was available from the reference subjects at examination A; at examination B, the SF level of ARGS ranged from 3.63 to 12.72 pmol/ml, with a mean of 7.19 pmol/ml (*P *= 0.78 compared with ARGS levels in subjects after meniscectomy at examination A).

**Figure 3:**
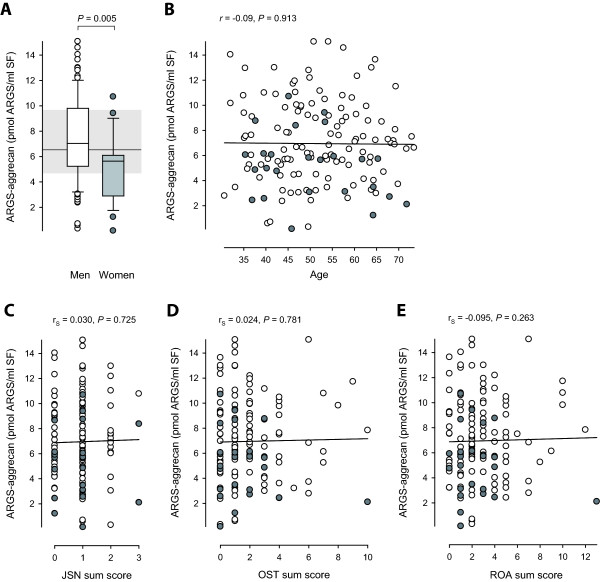
**Distribution of SF levels of ARGS-aggrecan by gender, age, joint space narrowing (JSN) sum score, osteophyte (OST) sum score, and the sum of the JSN and OST sum scores, termed radiographic OA (ROA) sum score**. Open boxes or circles represent men; grey boxes and circles represent women. **(a) **A box plot of SF ARGS in men (*n *= 116) and women (*n *= 25). The ends of the boxes define the 25^th ^and 75^th ^percentiles, with a line at the median, error bars defining the 10^th ^and 90^th ^percentiles and circles for individual outliers. Shaded area with line defines the 25^th ^and 75^th ^percentiles and median of the non-operated reference group (*n *= 17). Group difference was assessed by ANCOVA adjusted for age, body mass index, and time between meniscectomy and examination A. **(b) **Regression of SF ARGS and age with Pearson's correlation coefficient (*r*). **(c **through **e) **Regression of SF ARGS and JSN, OST, and ROA sum scores with Spearman's rank order correlation coefficients (*r*_S_).

The SF ARGS concentration was higher in men compared with women, with mean (range) values of 7.34 (0.31 to 15.07) and 5.14 (0.15 to 10.71) pmol ARGS/ml, respectively (*P *= 0.002; Figure 3a). The difference remained significant when adjusted for age, BMI, and time between meniscectomy and examination A (*P *= 0.005). BMI was higher in men (*P *< 0.001), with mean (range) values of 27.0 (21.8 to 41.4) kg/m^2 ^compared with 23.9 (17.9 to 32.0) kg/m^2 ^in women. No correlation, however, was seen between SF ARGS and BMI. No other differences, including radiographic status at examination A, or progression thereof, were seen between men and women. SF ARGS showed no correlation with age (Figure 3b), or any of the radiographic outcome scores, alone (Figure 3c and 3d) or in combination (Figure 3e).

### SF ARGS and progression of radiographic features of OA

Independent of stratification for ROA at examination A, a trend was seen for decreased likelihood of progression of radiographic features with increasing SF ARGS levels, with a mean odds ratio of 0.91 per pmol/ml SF ARGS (Table 3). A weak but significant negative association was found with a decrease in likelihood of loss of joint space with 0.89 times per pmol/ml increase in SF ARGS (Table 3). This association was stronger in subjects without ROA, whereas no association remained in those with ROA at examination A (Table 3).

**Table 3:** Odds ratios (ORs) from logistic regression analyses of associations between the examination A ARGS-aggrecan levels in SF and progression of radiographic features of OA from examination A to examination B at 7.5 years

	Total sample		Stratified ± ROA at examination A			
		
	*n *= 141		-ROA, *n *= 63		+ROA, *n *= 78	
				
	OR	*P*	OR	*P*	OR	*P*
Loss of joint space	*0.89 (0.80-0.99)*	*0.029*	*0.77 (0.63-0.94)*	*0.012*	*0.95 (0.82-1.10)*	*0.47*
	0.89 (0.79-0.996)	0.043	0.77 (0.62-0.95)	0.016	0.96 (0.81-1.13)	0.60

Osteophyte progression	*0.96 (0.87-1.07)*	*0.48*	*0.90 (0.76-1.07)*	*0.23*	*1.01 (0.88-1.15)*	*0.94*
	0.97 (0.87-1.08)	0.59	0.92 (0.76-1.11)	0.38	0.99 (0.86-1.15)	0.93

ROA progression	*0.90 (0.80-1.00)*	*0.059*	*0.87 (0.74-1.02)*	*0.092*	*0.91 (0.77-1.08)*	*0.30*
	0.89 (0.78-1.02)	0.10	0.87 (0.72-1.04)	0.13	0.90 (0.73-1.13)	0.37

Outcomes (loss of joint space, osteophyte progression, and ROA progression) are based on progression from examination A to examination B of the scores of joint space narrowing (JSN), osteophytes, and either JSN or osteophytes or both, scored according to the OARSI atlas [27]. OR and *P *values are crude (*italics*) or adjusted for age, gender, BMI, and time between examinations A and B (plain text), with 95% confidence interval in parentheses.

## Discussion

In this cohort of subjects with meniscectomy performed some 18 years earlier, we found SF ARGS levels no different from those in a reference group (without meniscectomy), and with no difference between subjects with ROA and subjects without. We further found that, within these relatively low and seemingly normal levels, SF ARGS and loss of joint space seem to be associated. However, contrary to our hypothesis, the association is negative: low levels of SF ARGS are associated with increased risk of loss of joint space.

We have reported, in acute inflammatory arthritis and early after injury, extremely elevated levels of SF ARGS, with a fold increase compared with healthy knee references of between 34 and 177, as measured with quantitative Western blot or ELISA, respectively [10,12]. We concluded that the best underlying explanation was an increased aggrecanase activity toward the interglobular domain (IGD) of aggrecan molecules that, to a large extent, were already C-terminally truncated and that the contribution from newly synthesized full-length aggrecan was minor [10,12]. The chondroitin sulfate (CS) 846 epitope has been suggested to be present mainly on recently synthesized aggrecan [37], and it was shown to be elevated two- to threefold in SF up to 20 years after knee injury compared with those uninjured [38]. In the present study, a decreased risk of joint space loss was found in subjects with higher levels of SF ARGS. A possible explanation for this could be that the higher SF ARGS levels observed here reflect a tissue-repair response involving an increased synthesis of aggrecan, in combination with aggrecanase activity. This would explain both an increase in the release of ARGS-aggrecan into the synovial fluid, and a decreased risk of loss of joint space due to an, at least in part, successful incorporation of newly synthesized aggrecan in the tissue. The fact that the negative association between SF ARGS and loss of joint space is stronger in subjects without ROA present at the examination, further strengthens the explanation that higher SF ARGS levels indeed reflect a higher synthetic activity in these subjects. Measuring only the ARGS neoepitope, we are, however, unable to ascertain the source of aggrecan fragments being degraded, which could span everything from newly synthesized aggrecan not incorporated into a functional matrix to C-terminally truncated aggrecan resident in the cartilage for a long period [39]. To better understand this balance between synthesis and degradation, our data indicate that the ARGS neoepitope marker may have to be used in combination with a marker of aggrecan synthesis.

Although both the quality and the quantity of the joint cartilage are suggested to influence the SF levels of biomarkers [19], we found no correlation between SF ARGS and radiographic status at the time of sampling in this dataset. Inclusion of the JSN sum score at examination A (our best approximation of joint-cartilage quantity) as an adjustment in the logistic regression model, did not essentially change the results (data not shown). Although we cannot exclude that the clearance rate of matrix molecules from the joint cavity might influence the associations noted here, marker concentrations were measured long after trauma, and a steady state between markers released into the SF and markers cleared from the SF has likely occurred [40].

Multiple reports have been published on aggrecan release into SF in disease [18,19,21,38,41-43]. After knee injury, SF levels of aggrecan were initially much elevated, but with time declined toward levels seen in uninjured knees [18,38]. The methods used were, however, not specific for proteolytic neoepitopes, which limits the interpretation of the underlying processes causing the aggrecan release. Confirming previous results obtained by protein sequencing [44,45], we showed with Western blots that the majority of aggrecan fragments released into SF in disease are aggrecanase generated and carry the ARGS-neoepitope [12]. We further showed a strong positive association between SF ARGS and knee-joint disease including knee injury, in which greatly elevated levels early after injury declined over time, with 1-year marker levels approaching those observed in knee-healthy individuals [10]. The present study is the first to suggest that SF levels of aggrecanase-generated ARGS fragments are associated with radiographic progression of OA.

In the search for a biomarker able to predict progression of OA, sample accessibility, as well as the specificity of the marker for the joint, tissue, and molecule in question, must be taken into consideration. Joint fluid is more difficult to obtain than blood or urine, but markers in SF are more likely to reflect local joint biology compared with markers in blood or urine. The most probable source of cartilage markers in knee-joint fluid is the PF or TF joints. We therefore chose to consider radiographic progression of OA in both joints, knowing that mixed patellofemoral and tibiofemoral OA is common in this meniscectomized sample [24], and that catabolic products of the cartilage of both joints are released into the SF.

By using the BIPEDS (Burden of disease, Investigative, Prognostic of disease, Efficacy of intervention, Diagnostic of disease, and Safety of intervention) classification of OA biomarkers [14,46], we showed that SF ARGS can be categorized as a diagnostic marker for disease with the capability of distinguishing a knee injured up to 1 year after injury from knees of healthy controls [10]. Here we showed that 18 years after a knee injury involving a meniscectomy, SF ARGS does not discriminate subjects with ROA from subjects without, nor can it be classified as a burden of disease marker for radiographic status. However, the association between SF ARGS and loss of joint space indicates a potential for SF ARGS as a prognostic marker for JSN.

The ELCL assay here used to measure ARGS-aggrecan is novel, based on an ELISA using a similar approach of capturing aggrecan fragments by a commercial anti-human aggrecan antibody and detecting with the BC-3 antibody directed at the ARGS neoepitope [35]. By using the same capture antibody, which is reactive against both G1- and G2-containing aggrecan fragments, together with the anti-ARGS MAb OA-1 [12,30], we found highly similar results on the same human SFs. The assay is more sensitive than the previously used keratan sulfate capture OA-1 ARGS ELISA [10,30] and is better suited for analysis of SF samples low in ARGS, such as those analyzed herein. The transition from ELISA to the ELCL format reduced the required sample volume by fourfold.

## Conclusions

We found that concentrations of SF ARGS 18 years after meniscectomy were inversely associated with loss of joint space, where low levels of SF ARGS increased the risk for progression.

## Abbreviations

ADAMTS: a disintegrin and metalloproteinase with thrombospondin motifs; BIPEDS: the OA biomarker classification groups Burden of disease, Investigative, Prognostic of disease, Efficacy of intervention, Diagnostic of disease, and Safety of intervention; CS: chondroitin sulfate; CS1: CS-rich domain 1; CTX-II: C-telopeptides of type II collagen; ELCL: electrochemiluminescence; ELISA: enzyme-linked immunosorbent assay; HABR: hyaluronic acid-binding region; IGD: interglobular domain; JSN: joint space narrowing; MAb: monoclonal antibody; MSD: Meso Scale Discovery; OA: osteoarthritis; OR: odds ratio; PF: patellofemoral; ROA: radiographic OA; SF: synovial fluid; TF: tibiofemoral.

## Competing interests

The authors declare that they have no competing interests.

## Authors' contributions

SL developed and ran the ARGS ECLC assay, carried out the statistical analysis and interpretation of data, and drafted the manuscript. ME, one of the principal investigators in the original study of meniscectomy, read and scored the radiographs together with another investigator (Ludvig Dahl) and revised the manuscript. AS contributed in the development of the ARGS ECLC assay and revised the manuscript. LSL conceived the original study of meniscectomy, collected samples, and revised the manuscript. All authors participated in the design, interpreted results, and approved the final manuscript.
